# Grafting (S)-2-Phenylpropionic Acid on Coordinatively Unsaturated Metal Centers of MIL−101(Al) Metal–Organic Frameworks for Improved Enantioseparation

**DOI:** 10.3390/ma15238456

**Published:** 2022-11-27

**Authors:** Rui Zhao, Xueyan Bai, Wenhui Yang, Kun Fan, Haiyang Zhang

**Affiliations:** 1 School of Light Industry, Beijing Technology and Business University (BTBU), Beijing 100048, China; 2 Department of Biological Science and Engineering, School of Chemistry and Biological Engineering, University of Science and Technology Beijing, Beijing 100083, China

**Keywords:** chiral metal–organic frameworks, post-synthetic modification, enantioseparation, liquid chromatography, MIL−101(Al)@SiO_2_

## Abstract

Chiral metal–organic frameworks (cMOFs) are emerging chiral stationary phases for enantioseparation owing to their porosity and designability. However, a great number of cMOF materials show poor separation performance for chiral drugs in high-performance liquid chromatography (HPLC). The possible reasons might be the irregular shapes of MOFs and the low grafting degree of chiral ligands. Herein, MIL−101−Ppa@SiO_2_ was synthesized by a simple coordination post-synthetic modification method using (S)-(+)-2-Phenylpropionic acid and applied as the chiral stationary phase to separate chiral compounds by HPLC. NH_2_−MIL−101−Ppa@SiO_2_ prepared via covalent post-synthetic modification was used for comparison. The results showed that the chiral ligand density of MIL−101−Ppa@SiO_2_ was higher than that of NH_2_−MIL−101−Ppa@SiO_2_, and the MIL−101−Ppa@SiO_2_ column exhibited better chiral separation performance and structural stability. The binding affinities between MIL−101−Ppa@SiO_2_ and chiral compounds were simulated to prove the mechanism of the molecular interactions during HPLC. These results revealed that cMOFs prepared by coordination post-synthetic modification could increase the grafting degree and enhance the separation performance. This method can provide ideas for the synthesis of cMOFs.

## 1. Introduction

Chiral compounds, which exist in two forms, the R-enantiomer and S-enantiomer, exhibit identical physical and chemical properties. In the chiral microenvironment, one enantiomer may be active, but the other may show negative or toxic effects [1,2]. For example, S-hydrochloroquine and S-chloroquine have a higher response to SARS-CoV-2 when studying COVID-19 [3]. With the increasing research and development of chiral compounds, they have been widely applied in the fields of pharmacology, agriculture, flavors and life science [4,5,6]. In order to obtain a high efficacy of pure optical chiral compounds, the discrimination of enantiomers appears particularly necessary [7,8].

To date, many technologies for enantiomeric separation have been developed, such as enantioselective crystallization [9], membrane resolution [10,11], biokinetic resolution [12] and chromatography. Among them, chromatography technology attracts more attention owing to its simple operation and wide usage [13]. Among various chromatographic methods, HPLC serves as the most applicable enantioseparation strategy [14,15] due to its high efficiency and low cost [16]. It is well known that the enantiomeric recognition of chiral stationary phases (CSPs) is the key parameter that significantly influences the performance of enantioseparation. Therefore, many researchers pay more attention to developing novel CSPs. In recent decades, a variety of chiral stationary phases have emerged. Cellulose, cyclodextrin and other traditional materials were first applied as CSPs in the 1990s [17,18,19]. With the increasing demand for CSP separation performance, the development of porous materials is essential, such as metal–organic frameworks (MOFs) [20], covalent organic frameworks (COFs) [21] and porous organic polymers (POPs) [22]. These new materials are increasingly popular due to their porosity and large specific surface area. In particular, MOFs possess advantages such as adjustable channels, structural diversity, and good thermal and chemical stability [23,24,25]. Consequently, MOFs have become widely employed as chiral stationary phases [26,27].

In addition to the advantages mentioned above, cMOFs are attracting more attention as novel and advanced CSPs owing to their abundant recognition sites, including hydrogen bonds, π-π interactions, van der Waal forces, coordination bonds and others [24,28,29]. Moreover, they can also provide suitable chiral microenvironments by introducing chiral groups [30]. However, some scholars have proven that the irregular shapes and nonuniformity of cMOF crystals can lead to high column pressures and long retention times [24], which limit their applications in liquid chromatography [31]. The fabrication of shell–core structural CSPs can overcome these problems and exhibit high column efficiency [24,32,33].

Currently, there are many methods for preparing cMOFs, including direct synthesis, post-synthetic modification and chiral induction. The method of direct synthesis is simple, but the obtained cMOFs, named homochiral MOFs, have poor chemical stability, which is not favorable for their repeated use in HPLC. For chiral induction, choosing chiral inducing agents is a big challenge, though MOFs are stable. In terms of post-synthetic modification (PSM), the introduction of chiral ligands will not change the crystal structure, so the stability of post-modified MOFs is better than that of homochiral MOFs. Additionally, MOFs modified by different kinds of chiral ligands can obtain diverse structures. Owing to the above advantages, cMOFs synthesized by PSM are more suitable for chiral separation in the liquid phase [34,35,36,37]. In the PSM strategy, most researchers adopt covalent post-synthetic modifications, where the undecorated MOFs are mainly NH_2_−MOFs. However, Qian found that the stability of the NH_2_−MOF crystal was worse than that of the original MOFs [38]. It is worth noting that covalently modified cMOFs still have other disadvantages, for instance, the low grafting density of chiral groups. Zheng et al. raised this question and attempted a new solution, which was a tandem modification, to solve this problem [39,40,41]. In addition to the above shortcomings, the multi-step reaction and low synthetic yield of cMOFs still need to be resolved. In addition to covalent PSM, there is still another strategy of PSM, which is to decorate the unsaturated metal sites of MOFs with ligands via a coordination reaction [42]. When using this method, most researchers have employed pyridyl groups [43], amine groups [42] or carboxyl groups [44,45] to modify MOFs. Of these, carboxyl groups not only easily react with metal sites in one step but also can provide hydrogen-bonding interaction sites, which is considered an alternative strategy for coordination PSM. Moreover, the skeletons used for PSM are original MOFs, which provide better structural stability.

In this study, we developed a method of cMOF synthesis using MIL−101 through coordination post-synthetic modification and applied it as a chiral stationary phase. The cMOFs, MIL−101−Ppa@SiO_2_, were prepared by coordination coupling to introduce chiral ligands into the pores of MOFs. In order to evaluate the feasibility of this method, chiral NH_2_−MIL−101−Ppa@SiO_2_ was also prepared by covalent bonding. The two types of cMOFs were packed into columns to separate various racemates.

## 2. Materials and Methods

### 2.1. Chemicals and Materials

Silica microspheres (UniSil 5–120, particle size: 5 μm, pore size: 120 Å) were provided by Nano-Micro Technology (Suzhou, China). 2-Amino-terephthalic acid (99.0%), 3-aminopropyltriethoxysilane (98.0%) and aluminum chloride hexahydrate (99.0%) were purchased from Alfa Aesar Chemical Co., Ltd. (Shanghai, China). Succinic anhydride (99.0%) was from Beijing InnoChem Science & Technology Co., Ltd. (Beijing, China). Terephthalic acid (99.0%) was bought from Acros Organics (Geel, Belgium). Bromo-tris(pyrrolidino)-phosphonium hexafluorophosphate (98.0%) was obtained from Bide Pharmatech Ltd. (Shanghai, China). 4-Dimethylaminopyridine (99.0%) was from Macklin Biochemistry Technology Co., Ltd. (Shanghai, China). (S)-(+)-2-Phenylpropionic acid (98.0%) was provided by Energy Chemical (Shanghai, China). Ultrapure water (18.2 MΩ·cm) was prepared using a Milli-Q Advantage water purification system (Millipore, Burlington, VT, USA). All racemates were used for chiral separation by HPLC, consisting of ketoprofen, L-phenylglycinol, 2-amino-1,2-diphenylethanol, R-1-Phenyl-1,2-ethanediol (Adamas-beta), D, L-mandelic acid, (±)-naproxen (TCI), ibuprofen (Sigma-Aldrich, St. Louis, MO, USA), D, L-alpha-methylbenzylamine, D-phenylglycinol, ethyl-(S)-3-hydroxybutyrate, S-1-Phenyl-1,2-ethanediol (Macklin, Shanghai, China), R, S-phenylethanol, 1,1′-bi-2-naphthol (Energy Chemical, Shanghai, China) and ethyl-R-3-hydroxybutyrate (Ark-pharm, Arlington Heights, IL, USA). All of the chemicals and reagents used were at least of analytical grade.

### 2.2. Preparation of Chiral Stationary Phases

#### 2.2.1. Synthesis of SiO_2_-NH_2_

The original silica microspheres were activated first. Briefly, spherical silica gel (5.0 g) was placed in 100 mL of HCl solution (20%, *v*/*v*) and subjected to ultrasound treatments. The mixture was then stirred in a three-necked round-bottom flask at 90 °C for 3 h. The product was washed with ultrapure water to a neutral pH and vacuum-dried at 110 °C overnight.

Amino-functionalized SiO_2_ was obtained according to the method of Chen et al. [46]. Briefly, activated silica microspheres (5.0 g) were mixed in 200 mL of anhydrous ethanol and stirred at room temperature for 30 min. Then, 2.0 mL of 3-Aminopropyltriethoxysilane (APTES) was introduced dropwise into the mixture and stirred at 70 °C for 24 h. The obtained SiO_2_−NH_2_ microspheres were finally washed with anhydrous ethanol more than 3 times and vacuum dried at 70 °C for 10 h.

#### 2.2.2. Synthesis of MIL−101@SiO_2_

The SiO_2_ microspheres were first modified with −COOH before preparing MIL−101@SiO_2_ [47]. Succinic anhydride (25.0 g) was dissolved in 200 mL of DMF, and nitrogen was blown onto the mixture for 10 min to remove oxygen. The obtained SiO_2_−NH_2_ microspheres (5.0 g) were then dispersed into the above solution and reacted at room temperature for 24 h. The product (SiO_2_−COOH) was treated with DMF and anhydrous ethanol more than 3 times and dried overnight.

The construction of MIL−101@SiO_2_ was carried out according to the method reported by Bromberg et al. [48]. Briefly, 1.0 g of carboxylic silica spheres (SiO_2_−COOH) and aluminum chloride hexahydrate (AlCl_3_·6H_2_O) (1.2 mmol, 0.2897 g) were mixed in 300 mL of DMF and stirred at room temperature for 3 h. Subsequently, 299.034 mg of terephthalic acid (BDC) dissolved in 300 mL DMF was added dropwise to the mixture and heated to 130 °C for 48 h. The products were first centrifuged using DMF at 700 rpm to remove the MIL−101 crystal impurities and then washed with dichloromethane more than 3 times.

#### 2.2.3. Synthesis of NH_2_−MIL−101@SiO_2_

NH_2_−MIL−101 (Al) microspheres were prepared using the same synthesis method as that used for MIL−101 (Al) with slight alterations [48]. In brief, the organic linker was changed from BDC to 2-amino-terephthalic acid (amino-BDC), and the other experimental conditions were kept the same.

#### 2.2.4. Synthesis of MIL−101−Ppa@SiO_2_

First, 0.5 g of MIL−101@SiO_2_ and 18 mmol (2500 μL) S-2-Ppa were added to 50 mL of DMF and agitated at 100 °C for 9 h. The product was then cleaned using the same treatment procedure as that used for MIL−101@SiO_2_.

#### 2.2.5. Synthesis of NH_2_−MIL−101−Ppa@SiO_2_

NH_2_−MIL−101−Ppa@SiO_2_ was prepared according to the method of Bonnefoy et al. [49]. First, 2500 μL of S-2-Ppa was added to 20 mL of a dichloromethane solution of PyBrOP (18 mmol, 8.3914 g) and stirred at room temperature for 1 h. Then, NH_2_−MIL−101@SiO_2_ (0.5 g) and DMAP (36 mmol, 4.3981 g) dissolved in dichloromethane (30 mL) were added. The resulting mixture was stirred at room temperature for 4 days. These materials were washed with dichloromethane and vacuum dried.

### 2.3. Characterization

Scanning electron microscopy (SEM) was conducted on a JSM-7401F, 20 kV instrument (Jeol, Tokyo, Japan). Before observation, the samples were covered with gold to increase their conductivity. The infrared absorption spectra were taken on an Avatar 370 infrared Fourier transform spectrometer (Nicolet, Oshkosh, WI, USA). The powder X-ray diffraction (PXRD) data were collected on a Bruker D8 Focus diffractometer (Bruker, Karlsruhe, Germany). The surface area and porosity were recorded with the Quantachrome Autosorb IQ3 (Quantachrome, Boynton Beach, FL, USA) using N_2_ adsorption at 77 K. NMR samples were prepared in 2.5 mm NMR tubes, and liquid ^1^H NMR data were recorded on a Bruker AV 300 spectrometer (Bruker, Germany). Before ^1^H-NMR testing, MIL−101−Ppa@SiO_2_ was dissolved in a mixture of deuterated dimethyl sulfoxide (d6-DMSO) and 20% deuterated hydrochloric acid (DCl) in D_2_O (molar ratio 7:1) [50].

### 2.4. Column Packing

The packed column was prepared using a high-pressure slurry packing method. Before packing them into the column, the obtained materials were activated in dichloromethane for three days. The materials (0.5 g) were then dispersed in 50 mL of n-hexane/isopropanol (*v:v* = 95:5) under ultrasonication for 5 min and packed into a stainless-steel column (100 mm long × 2.1 mm i.d, IDEX CORPORATION, Lake Forest, IL, USA) using n-hexane/isopropanol (*v:v* = 95:5) under a pressure of 50 MPa for 50 min. The MIL−101−Ppa@SiO_2_ and NH_2_−MIL−101−Ppa@SiO_2_ packed columns were prepared following the same procedure. The packed column was conditioned/equilibrated with isopropanol at a flow rate of 0.1 mL·min^−1^ for 8 h before chromatographic experiments [51].

### 2.5. HPLC

HPLC analysis was performed on the Shimadzu series system with a Shimadzu LC-20AT pump and a Shimadzu SPD-20A UV detector (Shimadzu, Kyoto, Japan). In order to choose an appropriate eluent, various compositions of mobile phase systems were compared and optimized, such as methanol–water, n-hexane–isopropanol and n-hexane–dichloromethane. The working solutions of chiral compounds were prepared at a concentration of 1 mg·mL^−1^. All HPLC separations were carried out at a 40 °C temperature. The flow rate was 0.5 mL·min^−1^, and the injection volume was 2 μL.

### 2.6. Molecular Docking

**Scheme 1:**
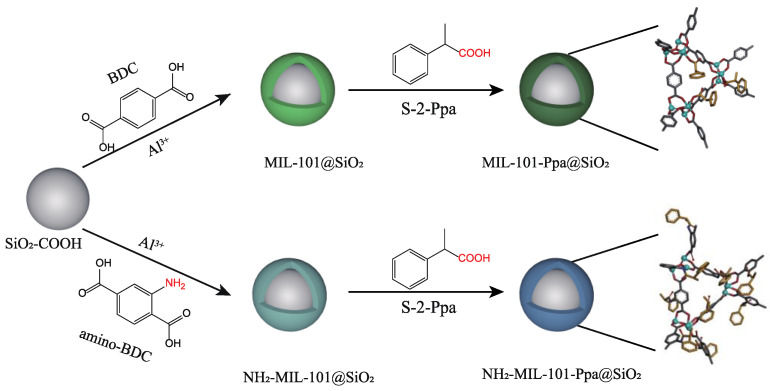
Schematic illustration of the structures of MIL−101−Ppa@SiO_2_ and NH_2_−MIL−101−Ppa@SiO_2_.

The interactions between modified MOFs (MIL−101−Ppa and NH_2_−MIL−101−Ppa) and racemates were investigated by molecular docking using the Autodock Vina 1.1.2 software [52]. Structural models of the modified MOFs were built on the basis of the MIL−101(Cr) crystal structure [53] via a modification of the metal-coordination network (MIL−101−Ppa) or the linker (NH_2_−MIL−101−Ppa), as illustrated in Scheme 1. MIL−101 has two types of mesoporous cages with diameters of ∼29 and 34 Å, formed by 20 and 28 hybrid super-tetrahedra (ST) building blocks, respectively. We chose two adjacent cages (denoted as ST20 and ST28) for modification, which were immersed in a cubic box of isopropanol solvent (one of the mobile phases in HPCL experiments) and then optimized via energy minimization using the GROMACS 2018 software [54] for subsequent docking calculations. The force-field parameters of the modified MOFs were generated by the OBGMX toolkit [55], and atomic charges of S-2-Ppa and linker groups were computed with the AM1-BCC charge model [56,57] using the “antechamber” tool [58]. The parameters of isopropanol were taken from previous work [59]. During docking, the search space was defined by the interior of the ST20 and ST28 cages and the channel between the two cages, and potential binding to the exterior of MIL−101 MOFs was blocked by isopropanol molecules. Such a task can be completed with the Visual Molecular Dynamics (VMD) software [60] by removing the solvent molecules in the search space from the energy-minimized structures. Note that there is no built-in parameter for the Al ion in the Autodock Vina software, and we used the Fe ion instead for a rough estimate. For each compound, docking was run 100 times with random seeds, and the best binding model with the lowest binding energy for each run was used for data collection.

## 3. Results and Discussion

In order to evaluate the performance of cMOFs@SiO_2_ prepared by coordination PSM, the MIL−101 crystal was deposited onto the surface of SiO_2_−COOH by solvothermal synthesis. (S)-(+)-2-Phenylpropionic acid (S-Ppa) was then grafted onto the unsaturated metal sites of MIL−101@SiO_2_ frameworks through a coordination reaction. This method provided a facile and short-time synthesis route to obtain chiral MIL−101−Ppa@SiO_2_. Moreover, NH_2_−MIL−101−Ppa@SiO_2_ was prepared by covalent PSM for comparison. The structures of MOF crystals and the general procedures for separation are illustrated in Scheme 1.

### 3.1. Characterization

**Figure 1:**
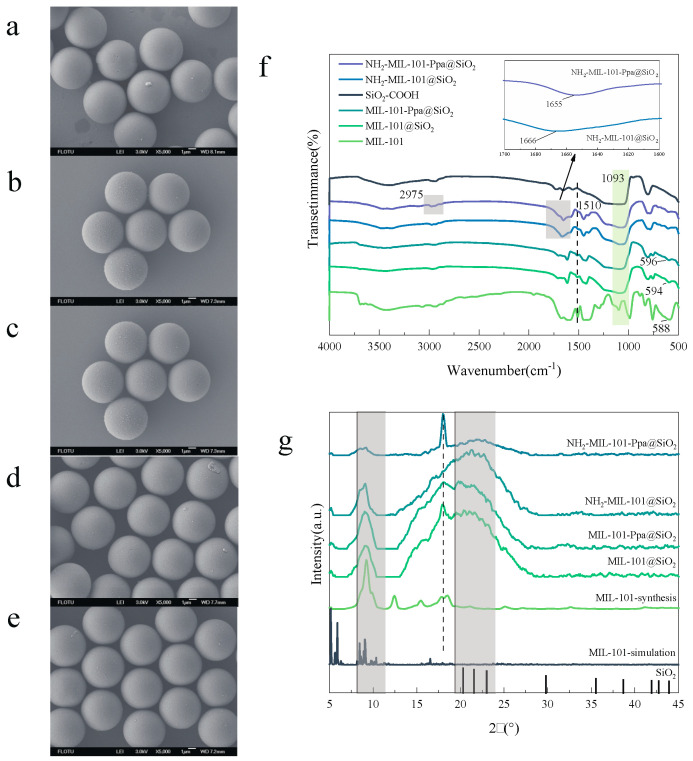
The SEM images of SiO_2_−COOH (**a**), MIL−101@SiO_2_ (**b**), MIL−101−Ppa@SiO_2_ (**c**), NH_2_−MIL−101@SiO_2_ (**d**) and NH_2_−MIL−101−Ppa@SiO_2_ (**e**); the FT-IR spectra of SiO_2_−COOH, MIL−101@SiO_2_, MIL−101−Ppa@SiO_2_, NH_2_−MIL−101@SiO_2_, NH_2_−MIL−101−Ppa@SiO_2_ and MIL−101 (**f**); the synthesized XRD patterns of NH_2_−MIL−101−Ppa@SiO_2_, NH_2_−MIL−101@SiO_2_, MIL−101−Ppa@SiO_2_, MIL−101@SiO_2_, MIL−101 and SiO_2_−COOH; and simulated XRD pattern of MIL−101 (**g**).

The morphologies of SiO_2_−COOH, MIL−101@SiO_2_, NH_2_−MIL−101@SiO_2_, MIL−101−Ppa@SiO_2_ and NH_2_−MIL−101−Ppa@SiO_2_ materials were characterized by Energy-Dispersive Spectroscopy (EDS) mapping and scanning electron microscopy (SEM). In the EDS mapping images of MIL−101@SiO_2_ and NH_2_−MIL−101@SiO_2_, the element Al was homogeneously dispersed in silica, which proved the formation of both MOFs@SiO_2_ composites (Figure S1). In addition, SEM was used to further observe the morphology of MOFs@SiO_2_. As shown in Figure 1a–e, all of the materials showed good dispersity and a regular shape. As shown in Figure 1a, the SiO_2_−COOH microspheres had a smooth surface with an average diameter of about 5 μm. After the immobilization of MIL−101 on the surface of SiO_2_ microspheres, MIL−101@SiO_2_ presented a rough surface. The average diameter of MIL−101@SiO_2_ increased to 5.2 μm (Figure 1b), which indicated the successful synthesis of MIL−101@SiO_2_, and the shell thickness was about 200 nm. Though a similar rough surface of NH_2_−MIL−101@SiO_2_ could be observed, the shell thickness of NH_2_−MIL−101 (140 nm) was a little thinner than that of MIL−101@SiO_2_ (Figure 1d). It is presumed that the growth of the MIL−101@SiO_2_ crystal was better than that of NH_2_−MIL−101@SiO_2_. Figure 1c,e shows the images of MIL−101−Ppa@SiO_2_ and NH_2_−MIL−101−Ppa@SiO_2_ morphologies. Few changes were observed after modification, which suggested that the post-synthetic modification with Ppa might not affect the morphologies of MOFs.

Fourier-transform infrared spectroscopy (FT-IR) was used to further confirm the preparation of MIL−101@SiO_2_, NH_2_−MIL−101@SiO_2_, MIL−101−Ppa@SiO_2_ and NH_2_−MIL−101−Ppa@SiO_2_ materials, as shown in Figure 1f. In the spectrum of SiO_2_−COOH, the peak intensity at 1093 cm^−1^ was ascribed to the vibration of Si-O-Si [32]. An adsorption peak at 1510 cm^−1^ in the spectrum of MIL−101 was attributable to the asymmetric and symmetric vibrations of the benzene group in MIL−101 [61]. These characteristic peaks of SiO_2_−COOH and MIL−101 could be observed in the spectrum of MIL−101@SiO_2_, which indicated that the MIL−101@SiO_2_ composite was formed [32]. Comparing MIL−101@SiO_2_ with MIL−101−Ppa@SiO_2_, the peak of the Al-O stretch slightly shifted from 594 cm^−1^ to 596 cm^−1^, which resulted from the introduction of Ppa to MIL−101 via coordination coupling [62]. In the spectrum of NH_2_−MIL−101@SiO_2_, the appearance of the characteristic band at 1666 cm^−1^ was due to the -NH_2_ linkage, which proved the successful synthesis of NH_2_−MIL−101 [63]. Evidence that NH_2_−MIL−101 was deposited onto the SiO_2_ microspheres could also be obtained from the spectrum of NH_2_−MIL−101@SiO_2_ in the same way as MIL−101@SiO_2_. Comparing the spectrum of NH_2_−MIL−101@SiO_2_ with that of NH_2_−MIL−101−Ppa@SiO_2_, the characteristic peak at 2975 cm^−1^ originating from the N-H bond of NH_2_−MIL−101 was increased, suggesting that the amide condensation reaction occurred between Ppa and NH_2_−MIL−101@SiO_2_. A characteristic peak appeared at 1655 cm^−1^ (NH_2_−MIL−101−Ppa@SiO_2_), which was attributed to symmetric stretching vibrations of −C=O in amide groups [64].

The powder X-ray diffraction (PXRD) patterns of the materials are shown in Figure 1g. As can be seen, a pronounced peak in the range of 20–25° corresponding to the spectrum of SiO_2_ appeared in the pattern of MIL−101@SiO_2_ [32], which proved the successful growth of MIL−101 on the SiO_2_ microspheres. According to the pattern of NH_2_−MIL−101@SiO_2_, the same evidence could be obtained to demonstrate the successful preparation of NH_2_−MIL−101@SiO_2_ composites. In addition, characteristic peaks at 2θ = 9.23° and 18° with high intensity belonged to MIL−101 crystals [65]. The same signal could be observed in the PXRD pattern of MIL−101@SiO_2_ and NH_2_−MIL−101@SiO_2_, which also indicated the successful synthesis of MOFs@SiO_2_ composites. However, the characteristic peaks of NH_2_−MIL−101@SiO_2_ were wider and weaker than those of MIL−101@SiO_2_, which indicated the poorer crystallinity of NH_2_−MIL−101@SiO_2_. This broad Bragg reflection of NH_2_−MIL−101@SiO_2_ might result from the small size effect [66]. Comparing the crystal peaks before and after modification, MIL−101−Ppa@SiO_2_ and NH_2_−MIL−101−Ppa@SiO_2_ had no new diffraction peaks, so it was assumed that Ppa did not influence the crystal structure of MOFs.

**Figure 2:**
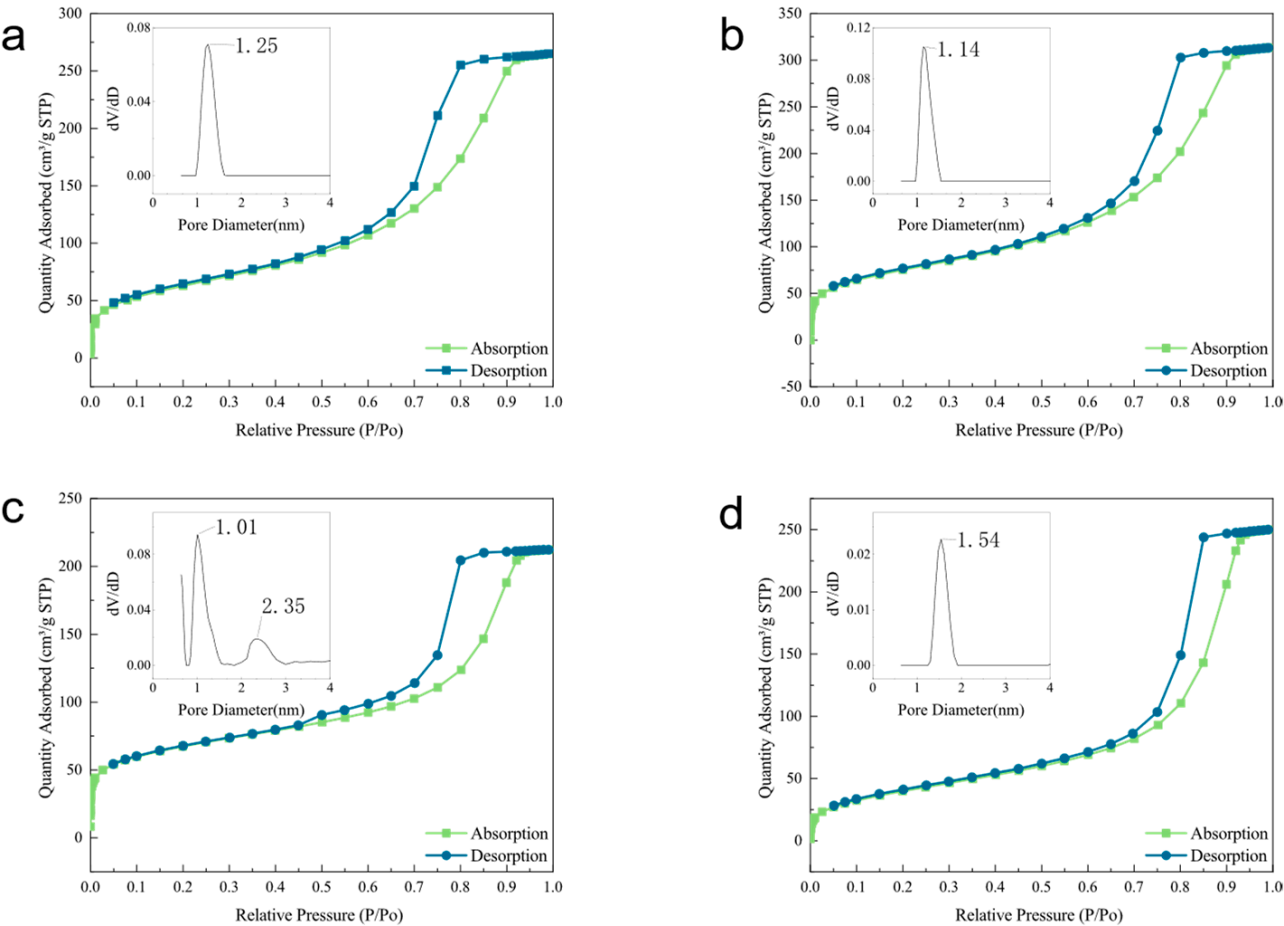
N_2_ adsorption isotherms and pore size distributions of MIL−101−Ppa@SiO_2_ (**a**), MIL−101@SiO_2_ (**b**), NH_2_−MIL−101@SiO_2_ (**c**) and NH_2_−MIL−101−Ppa@SiO_2_ (**d**).

The nitrogen adsorption–desorption isotherms of MIL−101@SiO_2_, MIL−101−Ppa@SiO_2_, NH_2_−MIL−101@SiO_2_ and NH_2_−MIL−101−Ppa@SiO_2_ exhibited the type IV isotherm pattern (shown in Figure 2a–d), which indicated the presence of mesopores and micropores due to MIL−101 (Al). Recently, our group found that mesopores and micropores formed when a thin shell of MOF crystals formed on SiO_2_ [67]. After modification, the adsorption isotherm classification did not change. The specific surface areas of NH_2_−MIL−101@SiO_2_ and NH_2_−MIL−101−Ppa@SiO_2_ were 240.541 and 147.162 m^2^·g^−1^, respectively. For NH_2_−MIL−101@SiO_2_ in Figure 2c, there are two kinds of pores. The pore sizes of NH_2_−MIL−101@SiO_2_ were 1.01 nm and 2.35 nm, and the corresponding fractions of pores were 0.094 and 0.020. After modification with Ppa, the pore size of NH_2_−MIL−101−Ppa@SiO_2_ decreased. According to the fraction of pores, it could be deduced that the size of the bigger pores declined from 2.35 nm to 1.54 nm, and the size of the smaller ones could not be detected. For MIL−101@SiO_2_ and MIL−101−Ppa@SiO_2_, the BET surface area decreased from 269.007 m^2^·g^−1^ (MIL−101@SiO_2_) to 225.994 m^2^·g^−1^ (MIL−101−Ppa@SiO_2_). Meanwhile, the average pore size was reduced from 1.25 nm to 1.14 nm, which indicated that the chiral ligand occupied the channel space but might not destroy the crystal structure. This is in good agreement with the results of FTIR and XRD, which further confirmed that MIL−101@SiO_2_ had better stability.

In order to obtain the grafting degree of MIL−101−Ppa@SiO_2_, ^1^H NMR analysis was performed. The NMR spectrum shows obvious peaks of BDC and Ppa in Figure S2. According to Yan’s and Ma’s reports, the peaks around 7.95 and 7.18 ppm were assigned to the hydrogen signals of the BDC and Ppa ligands, respectively [68,69]. The grafting ratio was defined as the ratio of the weights of grafted chiral ligands to the weights before grafting [70]. According to this definition, the grafting degree of MIL−101−Ppa@SiO_2_ was about 22.92%, which proved that Ppa was successfully modified. Furthermore, this grafting degree was higher than that of NH_2_−MIL−101−Ppa reported by Yan’s synthesis [69].

### 3.2. HPLC Separation of Racemic Compounds

**Figure 3:**
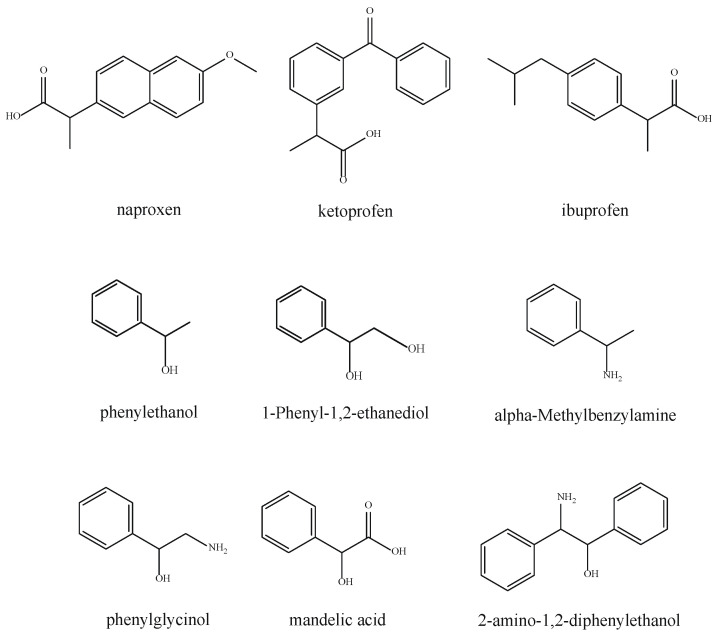
The structures of the selected chiral drugs and intermediates.

**Figure 4:**
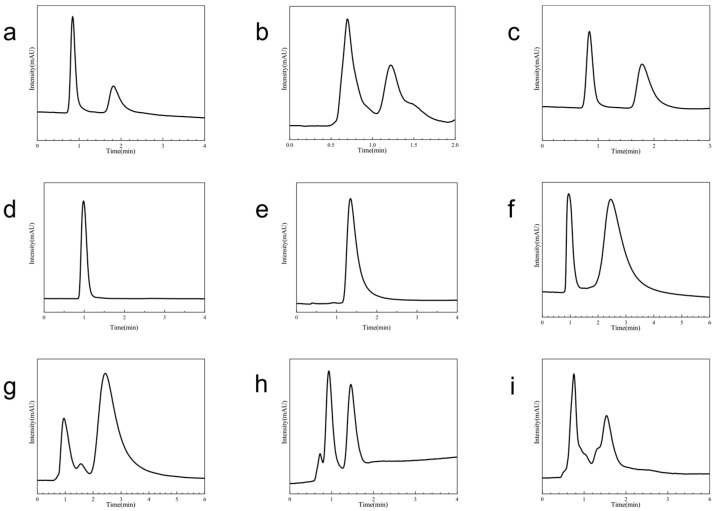
The chromatograms of (±)-naproxen (**a**), ketoprofen (**b**), ibuprofen (**c**), R, S-phenylethanol (**d**), R, S-1-Phenyl-1,2-ethanediol (**e**), DL-alpha-methylbenzylamine (**f**), DL-phenylglycinol (**g**), (±)-mandelic acid (**h**) and 2-amino-1,2-diphenylethanol (**i**) on the MIL−101−Ppa@SiO_2_ column.

In order to investigate the chiral separation ability of the MIL−101−Ppa@SiO_2_ CSPs, various types of racemic compounds were employed as targets. Herein, nine racemic compounds were used: rac-ketoprofen, (±)-naproxen, rac-ibuprofen, R, S-phenylethanol, R, S-1-Phenyl-1,2-ethanediol, (±)-mandelic acid, DL-alpha-methylbenzylamine, DL-phenylglycinol and 2-amino-1,2-diphenylethanol racemates, which were derived from non-steroidal drugs, phenylethanol and its derivatives and are widely used in the pharmaceutical industry, the cosmetic industry, protein engineering and biological chemistry [71,72,73,74]. The structures of these chiral compounds are shown in Figure 3. The separation results of all racemic compounds after optimizing the chromatographic mobile phase conditions are shown in Figure 4 and Table 1. The baseline separation of naproxen and ibuprofen with corresponding symmetric peak shapes was achieved, which exhibited the high enantioselectivity and good separation performance of the MIL−101−Ppa@SiO_2_ packed column. The common structural feature of these enantiomers was that they all possessed hydroxyl groups, which might be capable of hydrogen-bonding interactions with the carboxyl of MIL−101−Ppa@SiO_2_ [75]. Additionally, other possible interactions, including van der Waals forces, hydrophobic interactions and π-π interactions, might also contribute to the chiral recognition among the chiral target compounds and the MIL−101−Ppa@SiO_2_ column. For the other enantiomers, including ketoprofen, mandelic acid, alpha-methylbenzylamine, DL-phenylglycinol and 2-amino-1,2-diphenylethanol, complete separation could not be achieved. According to the structures of these racemic compounds, it was assumed that hydrogen-bonding interactions, π-π interactions and hydrophobic interactions might occur between chiral compounds and CSPs. Among them, carboxyl groups or amido groups supplied such significant hydrogen-bonding interaction sites in their structures that both the R-enantiomer and S-enantiomer showed strong retention behavior on the MIL−101−Ppa@SiO_2_ column. The peaks of two enantiomers were more likely to overlap, which led to low separation factors (Rs). Additionally, although R, S-phenylethanol and R, S-1-Phenyl-1,2-ethanediol had benzene ring and hydroxyl groups, they could not be separated due to their strong binding. In addition to the nine racemates, attempts were made to separate some other chiral compounds using the MIL−101−Ppa@SiO_2_ column, but they could not be isolated. For example, in Figure S3a, the chromatogram of 1,1′-bi-2-naphthol only has one peak owing to the steric effect. The molecular dimensions of 1,1′-bi-2-naphthol were larger than the pore size of MIL−101−Ppa@SiO_2_, so 1,1′-bi-2-naphthol could not enter the pores [28]. This illustrated that the size exclusion of MIL−101−Ppa@SiO_2_ played an important part, as well. DL-Ethyl-3-hydroxybutyrate does not have benzene rings and could not serve as an active hydrogen-bond donor (Figure S3b). Therefore, it was unable to be completely separated. According to the above results, the main mechanism of separation may be hydrogen-bonding interactions and π-π interactions.

In order to further evaluate the separation capacity of cMOFs, NH_2_−MIL−101−Ppa@SiO_2_ prepared by the covalent PSM method was applied to separate chiral compounds. As seen in Figure S4, only three compounds could be isolated on the NH_2_−MIL−101−Ppa@SiO_2_ column. Their factors (Rs) were similar to those of MIL−101−Ppa@SiO_2_. However, the others could not be separated using the NH_2_−MIL−101−Ppa@SiO_2_ column. The separation performance of the NH_2_−MIL−101−Ppa@SiO_2_ column was unsatisfactory. One of the possible reasons is that the exposure of the metal sites led to high electronegativity [76]. The other reasons were that the crystal forms of the original MOFs were better than those of NH_2_−MOFs.

### 3.3. Evaluation of Separation Performance

In order to evaluate the separation performance of the MIL−101−Ppa@SiO_2_ column, a comparison between the MIL−101−Ppa@SiO_2_ column and commercial column (Chiralpak OD column) was carried out. For the Chiralpark OD column, the separation factors (α) of naproxen, ibuprofen and ketoprofen were 1.03, 1.13 and 1.03, respectively [77], while the factors of the three above compounds on the MIL−101−Ppa@SiO_2_ column were 35.71, 21.98 and 10.02, respectively. The separation results showed that MIL−101−Ppa@SiO_2_ had better resolution and required less time. This confirmed that the MIL−101−Ppa@SiO_2_ column has the potential to be applied in the separation of these chiral compounds.

**Figure 5:**
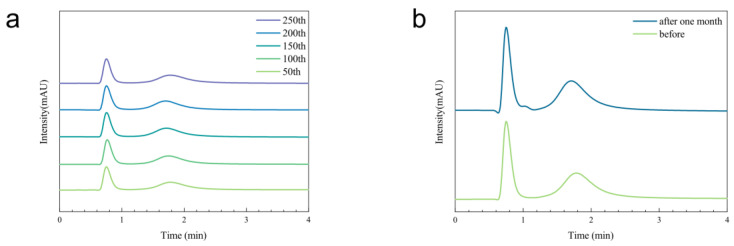
The chromatograms of DL-alpha-methylbenzylamine on the MIL−101−Ppa@SiO_2_ packed column after repeated injections: (1) 20th, (2) 40th, (3) 60th, (4) 80th and (5) 100th injection (**a**) and the injections before and after one month (**b**).

In addition, the reproducibility was tested by repeatedly separating alpha-methylbenzylamine on the MIL−101−Ppa@SiO_2_ column at 40 °C using n-hexane/isopropanol (10:90, *v:v*) as the mobile phase. There were no obvious changes in the 50th, 100th, 150th, 200th or 250th injections, which demonstrates the good reproducibility of MIL−101−Ppa@SiO_2_ after 250 times (Figure 5a). The relative standard deviations (RSDs, *n* = 5) of the retention time and peak area for replicate separations were 0.52% and 2.68%, respectively, which indicated the good reproducibility of the MIL−101−Ppa@SiO_2_ column, as well. After one month, alpha-methylbenzylamine could still be completely separated, with little change in the separation factor (Figure 5b).

**Figure 6:**
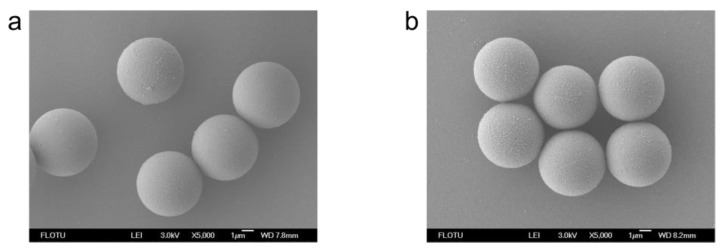
The SEM images of MIL−101−Ppa@SiO_2_ before (**a**) and after tests (**b**).

The stability of the MIL−101−Ppa@SiO_2_ column was characterized by SEM and PXRD, as well. Compared with the XRD spectrum of MIL−101−Ppa@SiO_2_ before packing the column, MIL−101−Ppa@SiO_2_ after separation still maintained its original crystal structure, as shown in Figure S5. The morphology of MIL−101−Ppa@SiO_2_ did not significantly change (Figure 6). This proved that MIL−101 retained its intact crystal on the surface of the support. The results above demonstrated that MIL−101−Ppa@SiO_2_ had good stability.

### 3.4. Docking Predictions

**Figure 7:**
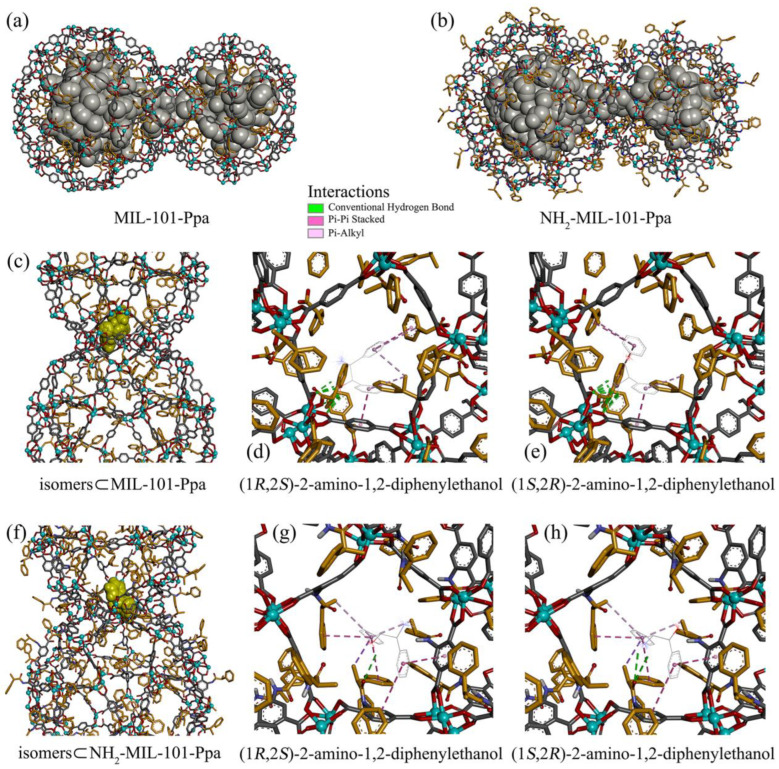
Two adjacent cages, ST28 (left) and ST20 (right), of MIL−101−Ppa (**a**) and NH_2_−MIL−101−Ppa (**b**) were used for docking calculations, where light-gray beads represent the search space for possible binding poses. Inclusion complexes of 2-amino-1,2-diphenylethanol isomers (space-filling models in yellow) with MIL−101−Ppa (**c**) and interaction networks between the binding partners for the R (**d**) and S (**e**) isomers. The structures in (**f**–**h**) are similar to (**c**–**e**) but for binding with NH_2_−MIL−101−Ppa. All pictures were generated by Biovia Discovery studio visualizer software. Ten representative binding poses are shown in panels (**d**–**h**) for a clear depiction of interaction networks. Al ions are shown with cyan balls. Carbon atoms in MIL−101 linkers are colored in gray, while they are orange in the Ppa group.

The interiors of two mesoporous cages (ST20 and ST28) and the channels between these two cages in MIL−101 MOFs were chosen as possible binding sites for the racemates, as indicated by the light-gray beads in Figure 7a,b. The binding affinities averaged over 100 independent docking runs for the *R* and *S* isomers are tabulated in Table 1. Due to the availability of reference standards, we could only determine the elution order of three racemates in HPLC experiments with MIL−101−Ppa and NH_2_−MIL−101−Ppa as the stationary phases. The *R* isomer of (±)-mandelic acid had a longer retention time than its *S* isomer, whereas the *R* isomer was eluted first with a shorter retention time compared to the *S* isomer of DL-phenylglycinol and 2-amino-1,2-diphenylethanol. A longer retention time indicates a stronger interaction (or binding affinity) with the stationary phase. Our docking calculations predicted binding affinities of ca. −8.0 and −8.4 kcal/mol for the binding of the *R* and *S* isomers of 2-amino-1,2-diphenylethanol to MIL−101−Ppa, in good agreement with the experimental elution order (Figure 4i and Table 1). Similarly, good agreement was obtained for the binding of 2-amino-1,2-diphenylethanol isomers to NH_2_−MIL−101−Ppa (Figure S4 and Table 1). For (±)-mandelic acid and DL-phenylglycinol, large errors for the predicted binding affinities precluded a clear-cut measurement, although the average values and/or the lowest binding affinities showed good agreement with the experiment (Table 1).

The racemates preferred to interact with the channel between the two adjacent cages of MIL−101 MOFs over the interiors of the cages, as shown in Figure 7c–h. This may be ascribed to the fact that the cage interior is surrounded only by aromatic groups, and additional interactions, such as hydrogen bonds, can be provided by the metal-coordination network and/or the modified MOF linkers. Hydrogen bonds, Pi-Pi stacking between aromatic rings, and Pi-alkyl hydrophobic interactions with the Ppa alkyl group are the driving forces responsible for binding (Figure 7d,e,g,h). Both the hydroxyl and amino groups of 2-amino-1,2-diphenylethanol were capable of hydrogen bonding with the carboxyl group of MOF linkers and the Ppa hydroxyl group. The *R* isomer of 2-amino-1,2-diphenylethanol used its hydroxyl group as a hydrogen-bond donor (Figure 7d), while its *S* isomer used the amino group. The amino group (Figure 7e,h) appears to form more hydrogen bonds with MIL−101 MOFs than the hydroxyl group (Figure 7d,g). Moreover, the *R* isomer had two Pi-Pi stacking and three Pi-Alkyl contacts with MIL−101−Ppa (Figure 7d), while the *S* isomer formed three Pi-Pi stacking and two Pi-Alkyl contacts. These findings led to a difference in the binding affinity between two isomers and MIL−101 MOFs. For NH_2_−MIL−101−Ppa, the modified linker was likely to produce steric hindrance, thereby preventing close contact with the metal-coordination networks (Figure 7g–h).

## 4. Conclusions

In this study, the enantioseparation performance of cMOFs prepared by two different post-synthetic modifications and the roles of MOFs and chiral ligands were investigated. Therein, MIL−101−Ppa@SiO_2_ was prepared via coordination coupling, and NH_2_−MIL−101−Ppa@SiO_2_ composites were synthesized by covalent bonding. According to the characterization results, MIL−101−Ppa@SiO_2_ had a larger surface area and higher grafting density than NH_2_−MIL−101−Ppa@SiO_2_. Moreover, as the chiral stationary phase, MIL−101−Ppa@SiO_2_ could separate more stereoselective drugs and intermediates within a shorter time and exhibited good chiral separation performance. According to the good reproducibility and stability, MIL−101−Ppa@SiO_2_ could be used for enantioseparation by HPLC. The molecular docking calculations also supported the experimental data and provide insights into the separation capacity of MIL−101−Ppa@SiO_2_. This work supplies a simple method to synthesize cMOFs and apply them as CSPs. Thus, this synthetic method may have more promising applications in the future.

## Figures and Tables

**Table 1:** HPLC separation of racemates on MIL−101−Ppa@SiO_2_ and NH_2_−MIL−101−Ppa@SiO_2_ columns with hexane/isopropanol as the mobile phase and molecular docking predictions ^*a*^.

Racemates	MIL−101−Ppa@SiO_2_					NH_2_−MIL−101−Ppa@SiO_2_				
	Mobile Phase (*v*/*v*)	R_s_ *^b^*	α *^c^*	Binding Affinity (kcal/mol) *^d^*		Mobile Phase (*v*/*v*)	R_s_*^b^*	α *^c^*	Binding Affinity (kcal/mol) *^d^*	
				R	S				R	S
Naproxen	40:60	1.61	35.71	−8.28 ± 0.09 (−8.3)	−8.37 ± 0.07 (−8.4)	-	-	-	−7.19 ± 0.03 (−7.2)	−7.00 ± 0.00 (−7.0)
Ketoprofen	60:40	0.92	10.02	−9.08 ± 0.10 (−9.2)	−9.26 ± 0.27 (−9.4)	-	-	-	−8.36 ± 0.07 (−8.5)	−8.28 ± 0.04 (−8.3)
Ibuprofen	40:60	1.62	21.98	−7.42 ± 0.04 (−7.5)	−7.58 ± 0.07 (−7.6)	-	-	-	−6.61 ± 0.03 (−6.7)	−6.60 ± 0.01 (−6.6)
1-Phenyl-1,2-ethanediol	-	-	-	−6.35 ± 0.11 (−6.6)	−6.45 ± 0.12 (−6.8)	-	-	-	−6.28 ± 0.36 (−6.7)	−6.25 ± 0.43 (−6.7)
Phenylethanol	-	-	-	−6.30 ± 0.02 (−6.4)	−6.39 ± 0.02 (−6.4)	-	-	-	−6.26 ± 0.39 (−6.5)	−6.42 ± 0.36 (−6.6)
α-Methylbenzylamine	10:90	1.08	12.71	−6.30 ± 0.00 (−6.3)	−6.40 ± 0.00 (−6.4)	20:80	0.88	10.38	−6.11 ± 0.41(−6.5)	−6.23 ± 0.36 (−6.5)
DL-Phenylglycinol	10:90	0.97	6.26	−6.36 ± 0.10 (−6.7)	−6.31 ± 0.10 (−6.8)	-	-	-	−6.04 ± 0.50 (−6.7)	−6.08 ± 0.51 (−6.7)
(±)-Mandelic acid	20:80	0.93	3.02	−6.71 ± 0.19 (−7.2)	−6.66 ± 0.16 (−6.9)	20:80	1.09	9.81	−6.25 ± 0.56 (−6.9)	−6.13 ± 0.49 (−6.9)
2-Amino-1,2-diphenylethanol	80:20	1.15	7.68	−8.02 ± 0.05 (−8.2)	−8.37 ± 0.05 (−8.4)	90:10	1.04	1.80	−7.70 ± 0.00 (−7.7)	−7.90 ± 0.01 (−7.9)

^*a*^ UV detection wavelengths for naproxen, ibuprofen and α-methylbenzylamine are 280, 273 and 210 nm, respectively; it is 254 nm for other racemates. The racemates that could not be separated in HPLC experiments are marked by short dashes. ^*b*^ Separation resolution $R_{s}=\frac{2\times \left(t_{2}-t_{1}\right)}{1.7\times \left(W_{1,\frac{h}{2}}+W_{2,\frac{h}{2}}\right)}$, where *t*1 and *t*2 are the retention times, and $W_{1,\frac{h}{2}}$ and $W_{2,\frac{h}{2}}$ are the full widths at half maxima of the first and second eluted enantiomers, respectively. ^*c*^ Separation factor α=k2k1=(t2−t0)t0(t1−t0)t0, where *t*_0_ is the column void time determined by 1,3,5-tri-tert-butyl-benzene. ^*d*^ Binding affinities between the modified MIL−101 MOFs and chiral compounds (R/S isomers) from 100 replicates of docking calculations; the lowest binding affinities are given in parentheses.

## Data Availability

Not applicable.

## Supplementary Materials

The following supporting information can be downloaded at https://www.mdpi.com/article/10.3390/ma15238456/s1, Figure S1. The EDS mappings of MIL−101@SiO_2_ (a) and NH_2_−MIL−101@SiO_2_ (b); Figure S2. The ^1^H NMR spectrum of MIL−101−Ppa@SiO_2_; Figure S3. The chromatograms of 1,1′-bi-2-naphthol racemates (a) and DL-ethyl-3-hydroxybutyrate (b) on the MIL−101−Ppa@SiO_2_ column. Chromatographic conditions: 0.5 mL·min^−1^, 25 °C. Figure S4. The chromatograms of (±)-naproxen (a), (±)-ketoprofen (b), (±)-ibuprofen R, S-phenylethanol (d), R, S-1-Phenyl-1,2-ethanediol (e), DL-alpha-methylbenzylamine (f), DL-phenylglycinol (g), (±)-mandelic acid (h) and 2-amino-1,2-diphenylethanol racemates (i) on the NH_2_−MIL−101−Ppa@SiO_2_ column. Chromatographic conditions: 0.5 mL·min^−1^, 25 °C. Figure S5. The PXRD patterns of MIL−101−Ppa@SiO_2_ before (a) and after tests (b).
