# Supplementary material for: Grafting (S)-2-Phenylpropionic Acid on Coordinatively Unsaturated Metal Centers of MIL−101(Al) Metal–Organic Frameworks for Improved Enantioseparation
Source: Materials (Basel). 2022 Nov 27;15(23):8456. doi: 10.3390/ma15238456 (PMC9740726; doi:10.3390/ma15238456)
Supplement: Supplementary file 1 [file materials-15-08456-s001.zip › materials-2031875-supplementary.pdf]

Supporting Information

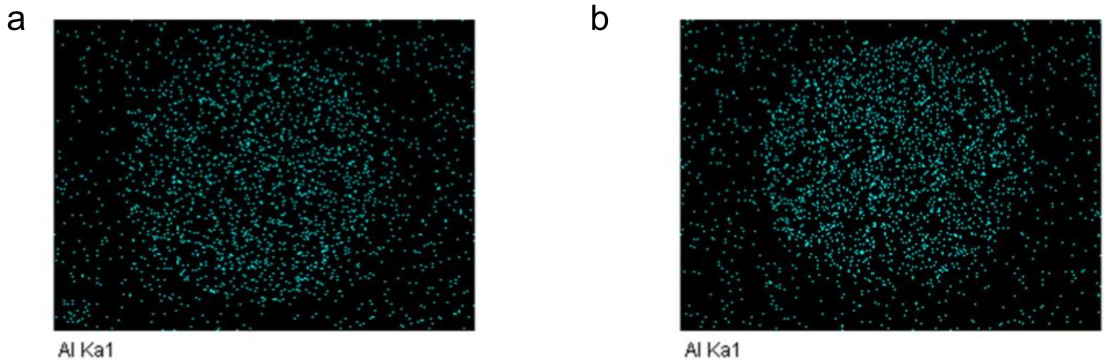

Figure S1. The EDS mappings of MIL-101@SiO_2(a) and NH_2-MIL-101@SiO_2(b).

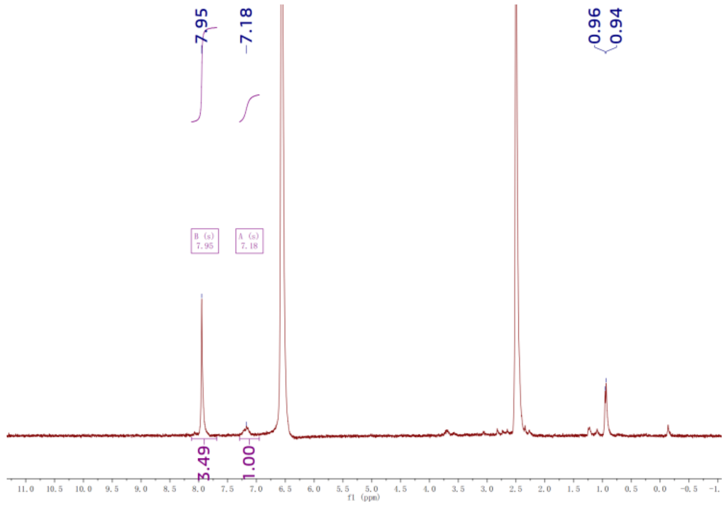

Figure S2. The ^{1}H NMR spectra of MIL-101-Ppa@SiO_2.

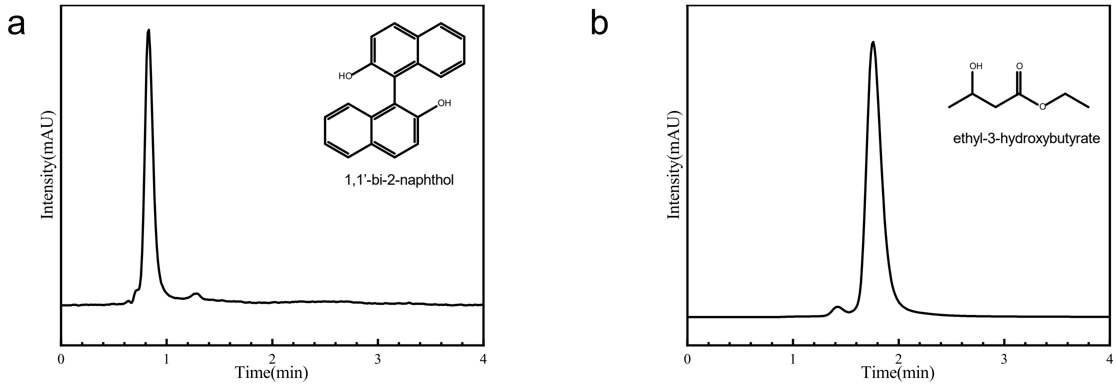

Figure S3. The chromatograms of 1,1'-bi-2-naphthol racemates(a), DL-ethyl-3-hydroxybutyrate (b) on the MIL-101-Ppa@SiO_2 column. Chromatographic conditions: 0.5 mL·min^{-1}, 25 °C.

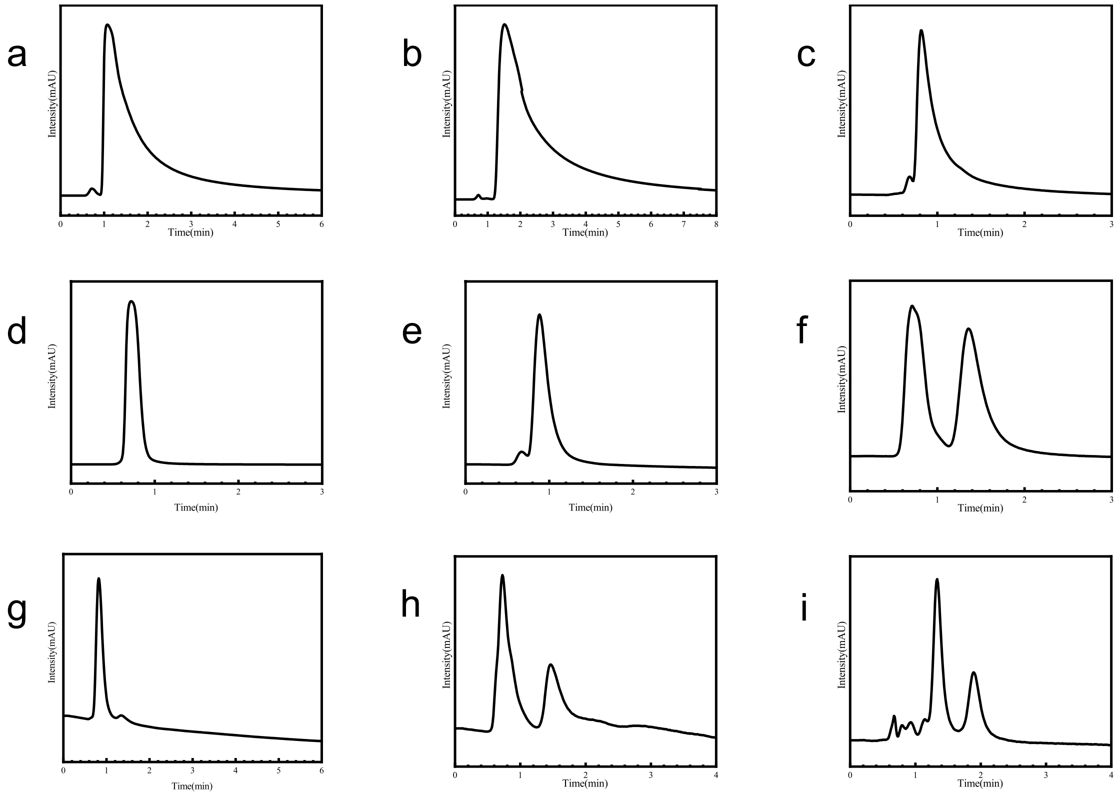

Figure S4. The chromatograms of (±)-naproxen (a), (±)-ketoprofen (b), (±)-ibuprofen R, S-phenylethanol (d), R, S-1-Phenyl-1,2-ethanediol (e), DL-alpha-methylbenzylamine (f), DL-phenylglycinol (g), (±)-mandelic acid (h), 2-amino-1,2-diphenylethanol racemates (i) on the NH_2-MIL-101-Ppa@SiO_2 column. Chromatographic conditions: 0.5 mL·min^{-1}, 25 °C.

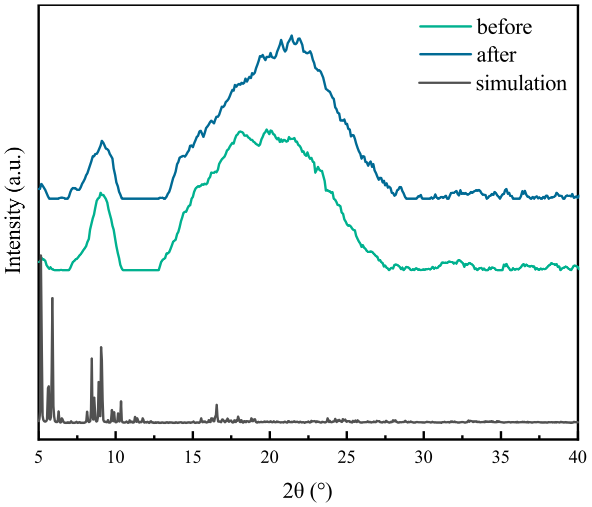

Figure S5. The PXRD patterns of MIL-101-Ppa@SiO_2 before(a) and after tests(b).
